# Massage-like stroking boosts the immune system in mice

**DOI:** 10.1038/srep10913

**Published:** 2015-06-05

**Authors:** Benjamin Major, Lorenza Rattazzi, Samuel Brod, Ivan Pilipović, Gordana Leposavić, Fulvio D’Acquisto

**Affiliations:** 1 William Harvey Research Institute, Barts and the London School of Medicine and Dentistry, Queen Mary University of London, London, UK; 2 Immunology Research Centre “Branislav Janković”, Institute of Virology, Vaccines and Sera “Torlak”, Belgrade, Serbia; 3 University of Belgrade-Faculty of Pharmacy, Department of Physiology, Serbia should be replaced by Department of Physiology, Belgrade, Serbia

## Abstract

Recent clinical evidence suggests that the therapeutic effect of massage involves the immune system and that this can be exploited as an adjunct therapy together with standard drug-based approaches. In this study, we investigated the mechanisms behind these effects exploring the immunomodulatory function of stroking as a surrogate of massage-like therapy in mice. C57/BL6 mice were stroked daily for 8 days either with a soft brush or directly with a gloved hand and then analysed for differences in their immune repertoire compared to control non-stroked mice. Our results show that hand- but not brush-stroked mice demonstrated a significant increase in thymic and splenic T cell number (p < 0.05; p < 0.01). These effects were not associated with significant changes in CD4/CD8 lineage commitment or activation profile. The boosting effects on T cell repertoire of massage-like therapy were associated with a decreased noradrenergic innervation of lymphoid organs and counteracted the immunosuppressive effect of hydrocortisone *in vivo*. Together our results in mice support the hypothesis that massage-like therapies might be of therapeutic value in the treatment of immunodeficiencies and related disorders and suggest a reduction of the inhibitory noradrenergic tone in lymphoid organs as one of the possible explanations for their immunomodulatory function.

Massage therapy is one of the many forms of rehabilitation medicine that has long been used as adjunct treatment for a variety of mental and physical conditions. Indeed, massage has been reported to ease muscle pain and favour repair^1^, to alleviate symptoms of nausea and fatigue in cancer patients^2^, to contribute to stress reduction and increase attention^1^,^3^ and to promote growth and development^4^. In recent times, massage has gained further popularity in clinical wards because of its applicability to a different range of patients from children^5^ to elderly^6^, professionals in the field of competitive sports^7^ and ‘non-active’ people from all walks of life^8^. Despite this wide range of applications, very little is known about the cellular and molecular mechanisms behind the beneficial effects of massage. Indeed, the lack of standardisation, the subjectivity of the receiver and the provider, together with the multiplicity of tissues and organs ‘activated’ by massage, has made this adjunct therapy a difficult practice to be studied in experimental terms.

The best way to describe the practice of massage is as a mechanical manipulation of body tissues by means of rhythmically applied strokes and pressure^9^. Following this definition, it is reasonable to think that one mechanism in which massage initiates its effects is through stimulation of the nervous system *via* different mechanoreceptors found in the skin (e.g. Meissner and Pacinian corpuscles, Merkel disc endings, Ruffini and free nerve endings)^10^. The pressure exerted on these receptors is then translated in different effects. More specifically, it can activate pathways that transmit signals along the long and myelinated nerve fibres to the lymbic area of the brain^11^ or can influence the release of soluble messengers such as substance P or serotonin that are considered responsible for mediating the ‘relaxing effects’ of massage^12^,^13^. Together these evidences suggest that emotional wellbeing and neuronal modulation are key elements for the therapeutic effectiveness of massage.

We are particularly interested in ways by which emotional wellbeing regulates the immune response^14^,^15^. Intrigued by recent clinical investigations that have explored the use of massage as mean of providing a combined positive effect on the emotional wellbeing and immune response^13^,^16^,^17^,^18^,^19^, we sought to set up an experimental system that would allow us to explore the immunomodulatory effects of massage-like therapy in mice. To this aim we tested the effects of stroking as surrogate for massage and we also compared human (finger-driven) and non-human (brush stroke-driven) approaches to assess two different types of stroking. Our results provide experimental evidence supporting the hypothesis that massage-like therapies have an immunomodulatory effect and suggest another possible cellular and molecular mechanism behind the therapeutic effects of this therapy.

## Materials and Methods

### Mice and chemicals

We used 5-week old C57BL/6 male mice purchased from Charles River for all experiments. Animals were housed in groups of maximum 6 mice per cage under specific-pathogen-free conditions and with free access to food and water. Mice were housed for 7 days prior to testing, making them 6 weeks old when the massage treatment began. All tests were conducted in a blinded fashion and according to the UK Animals (Scientific Procedures) Act, 1986. All experimental protocols were approved by the local biological service unit at Queen Mary University of London. Mice treated with hydrocortisone received a suspension of 2.5 mg of hydrocortisone hemisuccinate (Sigma-Aldrich) in 200 μl of PBS.

### Massage-like stroking therapy

Tests were performed double-blind during the light phase of the light-dark cycle. All efforts were made to minimize mouse discomfort in these behavioural experiments. Mice were brought to the testing room at least 30 minutes before the start of the test session to allow habituation to the testing environment. Unless otherwise specified, standard lighting (~50 lux) and quiet conditions were maintained throughout each experiment.

Mice were divided into three groups: 1) control (non-stroked) mice; 2) brush-stroked mice; 3) hand-stroked mice, each involving 6 mice per group, per experiment. Treatments were administered by placing a single mouse into a new cage every day as previously described^20^. These cages were identical to the home cage, however the lid, shredded paper, food and water were removed. New cages were used for each mouse to reduce odour from the previous mouse. Brush and hand stroked mice were stroked at a pressure of 100-150 mmH_2_O (or 7-11 mmHg) and at a speed of about 3 cm/sec according to a previously described protocol 21 whereas control mice were not touched (by hand or brush) throughout the 60-minute treatment. Brush stroking was applied using a No.5 da Vinci paintbrush while hand stroking was applied using three fingers of the preferred hand of the investigator as previously described^22^. For the hand-stroked treatment, the experimenter wore Bizzybee disposable vinyl gloves (Amazon, UK). These odourless gloves reduced human smell but maintained human contact i.e. warmth and pressure. Mice were stroked on the hairy skin found on the posterior dorsal thoracic and proximal hind limb^22^ in a cephalocaudal fashion (head to tail)^20^. Both brush and hand stroked mice received approximately 20 strokes every 5 minutes (0.5-1.0 Hz). Experiments were performed by 3 different experimenters trained to perform the same procedure. The reproducibility of the manual stimuli was tested and confirmed by similar application of pressure to a small balloon connected to a pressure gauge as previously reported 21.

### Open field activity test

The open field test (OFT) is an ethologically based paradigm that provides objective measures of exploratory behaviour as well as a valid initial screen for anxiety-related behaviour in rodents. The test was carried out as previously described 23. The apparatus consisted of a white PVC arena (50 cm × 30 cm × 20 cm) divided into 10 cm × 10 cm squares (n = 15). The 3 central squares defined the “centre” region. Each mouse was placed in a corner square, facing the wall, and observed and recorded for 3 minutes. The total number of squares crossed (all four paws in), total number of rears (defined as both front paws off the ground, but not as a part of grooming) and number of centre crossings was recorded. The walls and floor of the arena were thoroughly cleaned between each trial.

### Light-dark shuttle box

In this test, exploratory activity reflects the combination of hazard and risk avoidance^24^. The apparatus consisted of a 45 cm × 20 cm × 21 cm box, divided into two distinct compartments: one-third (15 cm long) painted black, with a black lid on top, the remaining two thirds painted white and uncovered. A 2.5 cm × 2.5 cm opening joined the two compartments. One side of the bright box was transparent to enable behavioural assessment and the averseness of this compartment was increased by additional illumination supplied by a 50 W lamp placed 45 cm above the centre of the box floor. The test was performed in accordance with a previous published protocol 25. Each mouse was placed in the bright compartment, facing away from the opening and allowed to explore the box for 5 minutes. Dependent variables included the time spent in the light area, latency to cross to the dark area (all four paws in) and the total number of transitions between compartments. The apparatus was cleaned after each trial.

### Fluorescence histochemistry and quantification of catecholamine-containing nerve fibres

Thymus and spleen were cut with a cryostat to obtain serial 16 μm thick sections and treated according to a modified version of the sucrose phosphate glyoxylic acid (SPG) method^26^ to identify cathecolamine-containing nerve fibres. Briefly, sections were first dipped in a solution containing 1% glyoxylic acid, 0.2 M sucrose, and 0.236 M potassium phosphate monobasic (pH 7.4), then drained and finally covered with non-auto fluorescent immersion oil, heated at 95 °C for 2.5 min. To prevent diffusion and photodecomposition of fluorescence, the sections were analysed and photographed on the same day using an Olympus BH 2 fluorescence photomicroscope (Olympus Optical Co. Ltd, Tokyo, Japan) equipped with exciter filter BG 12 and barrier filter Y495, Color View III digital camera (Olympus Soft Imaging Solutions GmbH, Münster, Germany) and AnalySIS FIVE software (Olympus Soft Imaging Solutions). The digital quantification of fluorescence intensity was performed using ImageJ software as described previously 27. Photomicrographs of 10 randomly chosen relevant test areas (×40 magnification) were taken in each of the 5 sections per thymus. After converting images to gray-scale mode, outlines of each fluorescent nerve profile were traced and intensities of fluorescent signal recorded (pixel intensity per unit area). To correct for the background fluorescence, the outlines were then placed on the closest adjacent areas not containing fluorescent material and the obtained values were subtracted from those corresponding to nerve profiles. The data were presented as fluorescence intensities of groups subjected to experimental treatment normalized to those of respective control groups.

### Noradrenaline concentration

Noradrenaline concentrations in spleen and thymus were determined using ELISA. The analysis was performed using a commercial ELISA kit (Labor Diagnostika Nord GmbH & Co., Nordhorn, Germany), according to the manufacturer’s protocol.

### Quantification of nerve fibre density

Fluorescent nerve fibre density in 10 randomly taken images (×40 magnification) from 5 thymic sections per animal was measured using a stereological grid point-counting approach^28^ and expressed as the percentage of field area occupied by the fluorescent nerve profiles.

### Flow cytometric analysis

Lymphocytes collected from lymphatic organs (e.g. thymus, spleen) were stained in 100μl of FACS buffer (PBS containing 5% FCS and 0.02% of NaN_2_). The antibodies used were anti-CD3 PE (clone 145-2C11, eBioscience), anti-CD4 FITC (clone GK 1.5, eBioscience), anti-CD8 Cy5 (clone 53-6.7, eBioscience), anti-CD25 FITC (clone PC61, BioLegend), anti-CD69 PE (clone H1.2F3, eBioscience). Cells were labeled with the appropriate concentration of conjugated antibodies for 1 h at 4 °C as previously described^29^. After labeling, cells were washed and analyzed. In all experiments stained cells were acquired with FACScalibur flow cytometer and analyzed using FlowJo^TM^ software (Tree Star, Inc., Oregon Corporation).

### T cell activation and cytokine production

Splenic T cells (1 × 10^5^ cells/200 μl) were incubated with medium alone or stimulated with the indicated concentration of plate-bound anti-CD3 and anti-CD28 in 96-well plates. For CD25 and CD69 upregulation, lymph node T cells were stimulated for about 16 hr while the supernatants of 24 hr culture were used for cytokine production. Cytokine production was measured by cytometric bead assay using the mouse Th1/Th2 10plex kits (eBioscience). Each sample (25 μl of cell culture supernatant) was incubated with 50 μl bead mixture and 50 μl mix of antibodies conjugated with biotin for 2 h. After two washes, PE-conjugated streptavidin was added and samples were left rocking for 1 hour in dark. Finally, samples were washed and stored overnight at 4 °C. Standards diluted serially for 7 times were prepared and processed at the same time. Samples were analysed using BD LSR Fortessa and the FlowCytomix software (eBioscience).

### Statistical analysis

Results were analysed as previously described^30^,^31^ using GraphPad. A one-way ANOVA followed by Bonferroni post-test or Newman-Keuls post-hoc comparisons was used for comparison between groups. Statistical significance was determined at p < 0.05. The results were expressed as mean ± S.E.M.

## Results

### Massage-like therapy does not cause changes in anxiety-like behaviour

**Figure 1:**
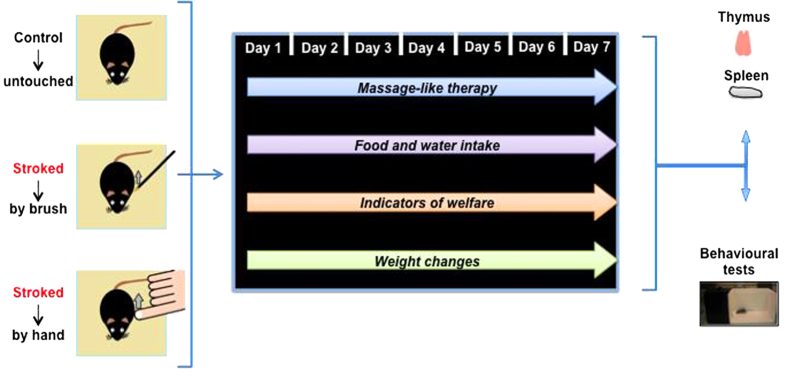
Massage-like stroking paradigm and associated procedures. The scheme in the figure summarizes the overall experimental procedure followed in this study. C57BL/6 male mice were divided in 3 groups of 6 mice and subjected to massage-like therapy (hand-stroked or brush-stroked) or left untouched (control) for 7 days as detailed in Materials and Methods. At the end of the treatment, mice were subjected to behavioural tests and analysed for their immune repertoire.

The scheme in Fig. 1 summarizes the experimental protocol we used for this study. We chose to administer the massage-like therapy for a period of 8-days in light of previous investigations, in which we assessed the effect of ‘behavioural modulators’ such as enriched environment on the immune system (unpublished data). In this and other similar experimental settings we found that changes start to occur and become significant at this time point.

**Figure 2:**
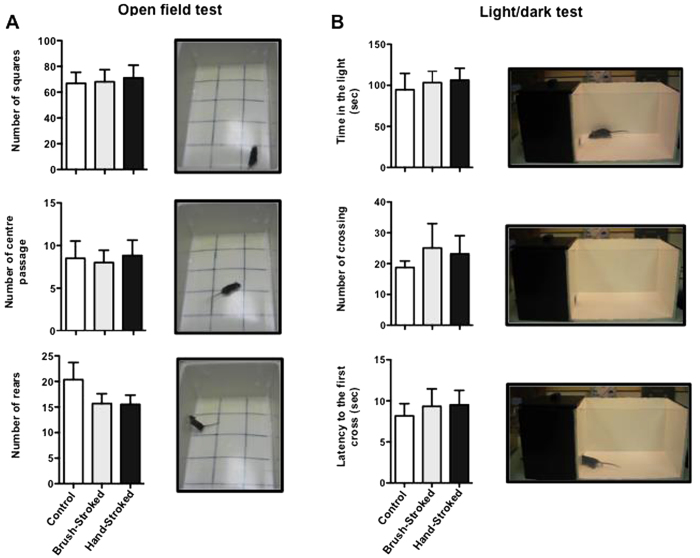
Massage-like stroking does not influence anxiety-like behavior. C57BL/6 male mice were subjected to massage-like therapy (hand-stroked or brush-stroked) or left untouched (control) for 7 days and then tested for their level of anxiety like-behaviour. (**A**) The bar graphs show the total number of squares crossed, centre crossings and rears recorded during a 5-minute session of the open field test. The pictures (from the top) show representative images of the square, centre crossing and rears, respectively. (**B**) The bar graphs show the time spent in the light, number of crossings and latency to the first cross recorded during a 5-minute trial in the light-dark shuttle box test. The pictures (from the top) show representative images of mouse in the light area, fully crossed (when the full four paws are in the dark side of the box) and at the first cross, respectively. Values are expressed as mean ± S.E.M. and representative of four experiments, each involving 6 mice per group.

Throughout the 8 days of massage-like therapy, we monitored indicators of welfare (http://www.nc3rs.org.uk/behaviour-laboratory-mice-indicator-welfare-state-genetically-modified-mice) such as weight change, coat condition, piloerection, freezing, number of faecal boli, food and water intake, and social aggressive behaviour. None of these measurements resulted in any significant difference (data not shown) between the 3 groups. We assessed further changes in behaviour and wellbeing, in particular anxiety-like behaviour, using two standard methods: the open field and the light-dark shuttle box. No differences in the number of squares, center crossings or rears were observed in the open field test among the three groups (Fig. 2A). Similarly, both brush- and hand-stroked mice showed no significant difference compared to control in the number of light/dark transitions or time spent in the lit area when tested with the light/dark shuttle box (Fig. 2B). Together these results suggested that massage-like stroking did not induce any significant changes in welfare, exploratory activity or anxiety like behavior.

### Massage-like therapy increases thymus cellularity and boosts the T cell repertoire

**Figure 3:**
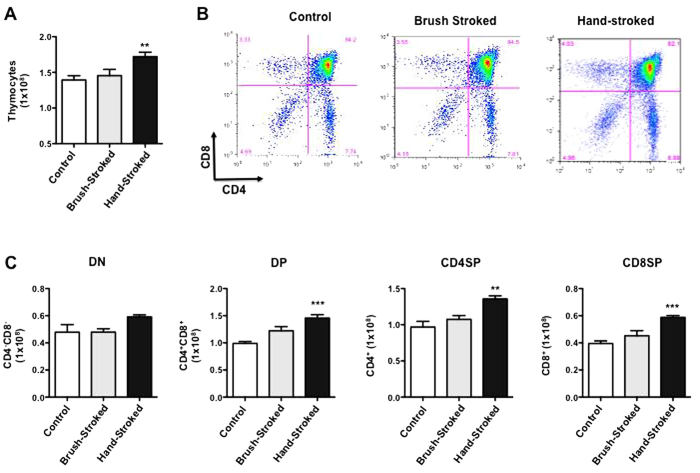
Massage-like stroking boosts T cell development. C57BL/6 male mice were subjected to massage-like therapy (hand-stroked or brush-stroked) or left untouched (control) for 7 days and then analysed for their T cell repertoire in the thymus. (**A**) The bar graph shows the total number thymocytes recovered at the end of the treatment while (**B**) shows representative dot plots of the different subpopulations of thymocytes: double negative CD4^-^CD8^-^ (DN; bottom left quadrant), double positive CD4^+^CD8^+^ (DP; top right quadrant), single positive CD4^+^ (CD4SP; bottom right quadrant), single positive CD8^+^ (CD8SP; top left quadrant). (**C**) Shows the absolute numbers of each subpopulation mentioned above. Values are expressed as mean ± S.E.M. and representative of four experiments, each involving 6 mice per group. ** p < 0.01; ***p < 0.001.

To assess the possible impact of massage-like therapy on the immune response we first tested its effects on T cell development in the thymus. The results showed an increase in thymocyte count in massaged mice in comparison to control non-stroked mice (Fig. 3A). Interestingly, significant differences were obtained only with hand-stroked mice (p < 0.01) but not with brush-stroked ones. This increase in cellularity was not T cell-lineage specific since FACS analysis of thymocytes for their CD4/CD8 profile showed only a slight difference between the CD4^+^/CD8^+^ cell percentages between control and brush- or hand-stroked mice (Fig. 3B). When considering the total number of each thymocyte subpopulation, only hand-stroked mice showed a significant increase in CD4^+^CD8^+^ double positive (DP) (p < 0.001), CD4^+^ single positive (SP) (p < 0.01), CD8^+^ SP cells (p < 0.001) compared to control (Fig. 3C), suggesting a possible effect of massage-like therapy on negative selection. Because brush-stroked mice showed no significant difference compared to control in any of the measures mentioned above, we continued our tests focusing on the effects of hand-massaged mice.

**Figure 4:**
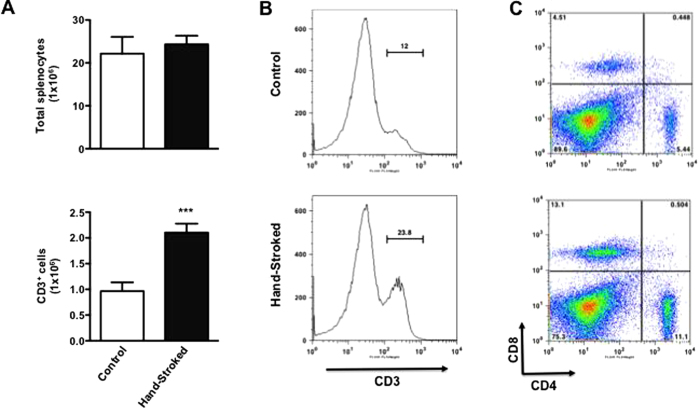
Massage-like stroking boosts the repertoire of mature T cells. C57BL/6 male mice were subjected to massage-like therapy (hand-stroked) or left untouched (control) for 7 days and then analysed for their T cell repertoire in the spleen. (**A**) The top bar graph shows the total number splenocytes recovered at the end of the treatment while the bottom shows the number of CD3^+^ T cells obtained after gating within splenocytes for this population. The two panels in (**B**) are representative histograms of the CD3^+^ gated population while those in (**C**) show their respective CD4/CD8 profile. Values are expressed as mean ± S.E.M. and representative of four experiments, each involving 6 mice per group. *** p < 0.001.

Performing the same phenotypic analysis of the thymus on splenic cell populations we observed a similar enhancing effect of massage on T cell cellularity. The total count of post-Ficoll cells (mainly mononuclear cells) did not show any significant differences between hand-stroked and control (Fig. 4A, top panel). However, gating on the CD3^+^ cells (Fig. 4B) and converting percentages in absolute numbers (Fig. 4A, bottom panel) revealed an almost 100% increase (p < 0.001) of T cells in the hand-stroked compared to control, respectively. As for the thymus, the increase in T cell population was not skewed towards either CD4^+^ or CD8^+^ cell lineages (Fig. 4C).

### Massage-like therapy reduces the noradrenergic tone of the thymus and spleen

Catecholamines released in lymphoid organs under the control of the central nervous system level are known regulators of the immune repertoire^32^,^33^. To test the possibility that cathecolamines mediated the boosting effects of massage-like therapy in our tests, we measured noradrenaline concentration and the fluorescence intensity and density of noradrenergic nerve fibres in both thymus and spleen.

**Figure 5:**
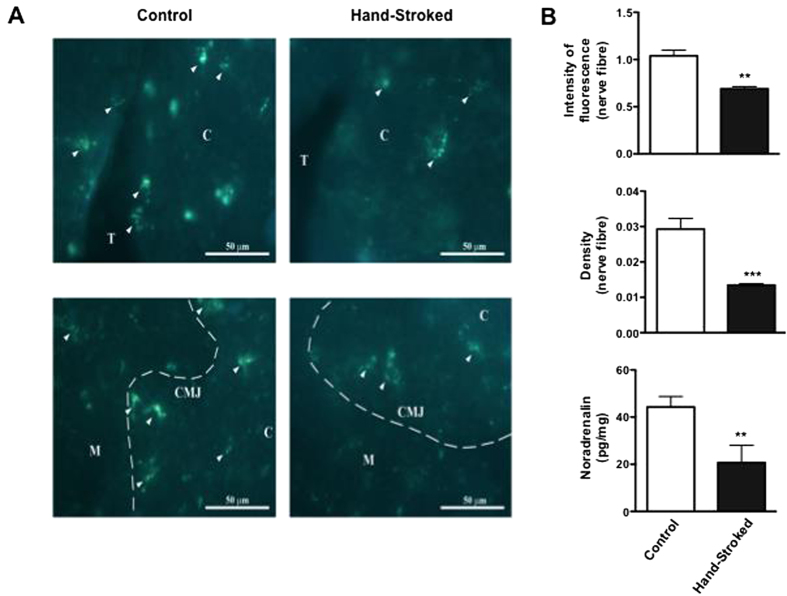
Massage-like stroking reduces the thymic noradrenergic tone. C57BL/6 male mice were subjected to massage-like therapy (hand-stroked) or left untouched (control) for 7 days and then analysed for their noradrenergic innervation and tone in the thymus. The immunofluorescence micrographs in (**A**) show representative images of the noradrenergic nerves present in different sections of the thymus: C = cortex; T = trabeculae; M = medulla; CMJ = corticomedullary junction. The bar graphs in (**B**) show (from the top) intensity of nerve fibre fluorescence, density of the nerve fibres (%) and noradrenaline concentration (pg/mg) in tissue homogenates. Values of each bar column are expressed as the mean ± S.E.M. of n = 6 mice. ** p < 0.01; ***p < 0.001.

Immunofluorescence analysis of thymus sections for catecholamine-containing nerve fibres showed a significant reduction in fluorescence intensity in both cortical and cortico-medullary junction areas of hand-stroked mice compared to control (Fig. 5A, right and left panels, respectively). This was consistent with the fact that large noradrenergic nerve plexuses predominate in the subcapsular cortex and corticomedullary junction, where more mature thymocytes reside^34^,^35^. Individual varicose nerve fibers branching from these plexuses terminate in the cortical and more rarely medullary parenchyma^34^,^35^. Quantitative analysis of both fluorescence intensity and nerve density confirmed these observations and showed a reduction of about 40% (p < 0.01) and 60% (p < 0.001), respectively, in hand-stroked tissue compared to control (Fig. 5B, top and middle panels, respectively). Finally, analysis of the noradrenaline content in tissue homogenates mirrored the immunofluorescence results and showed an overall reduction by about 50% (p < 0.01) of the noradrenaline levels in massaged mice compared to control (Fig. 5B, bottom panel).

**Figure 6:**
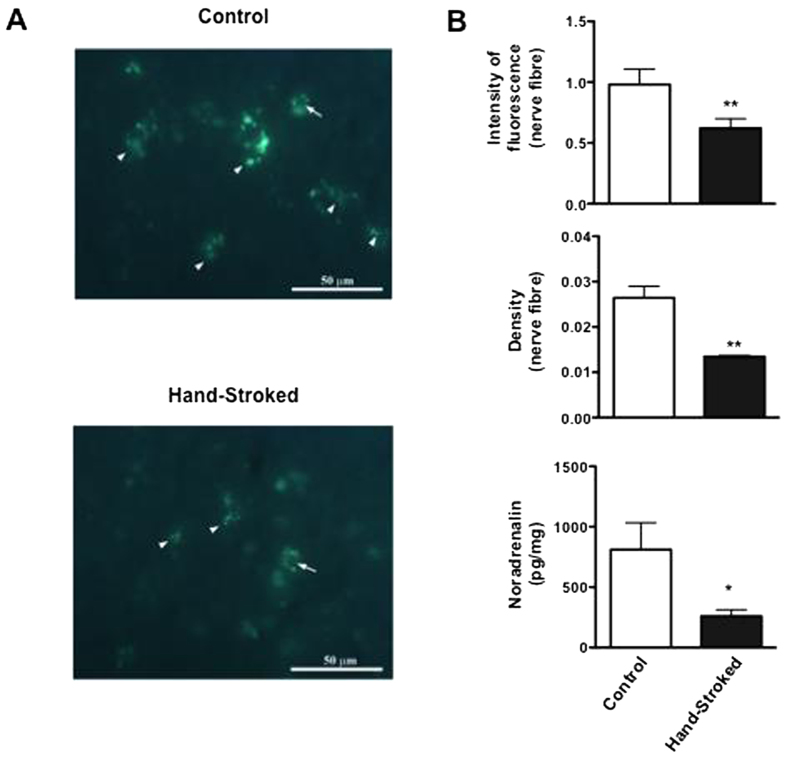
Massage-like stroking reduces the splenic noradrenergic tone. C57BL/6 male mice were subjected to massage-like therapy (hand-stroked) or left untouched (control) for 7 days and then analysed for their noradrenergic innervation and tone in the spleen. The immunofluorescence micrographs in (**A**) show representative images of the noradrenergic nerves present in different sections of the spleen. The bar graphs in (**B**) show (from the top) intensity of nerve fibre fluorescence, density of the nerve fibres (%) and noradrenaline concentration (pg/mg) in tissue homogenates. Values of each bar column are expressed as the mean ± S.E.M. of n = 6 mice. * p < 0.05; ** p < 0.01.

We performed the same analysis on the spleen and obtained similar results e.g. a reduction in the noradrenergic fibre fluorescence intensity (p < 0.01) and density (p < 0.01) (microphotographs in Fig. 6A and top and middle panels in 6B) and a significant reduction in noradrenaline levels in the spleen homogenates of hand-stroked mice compared to control (p < 0.05) (Fig. 6B, bottom panel).

### Massage-like therapy does not change the activation profile of mature T cells

**Figure 7:**
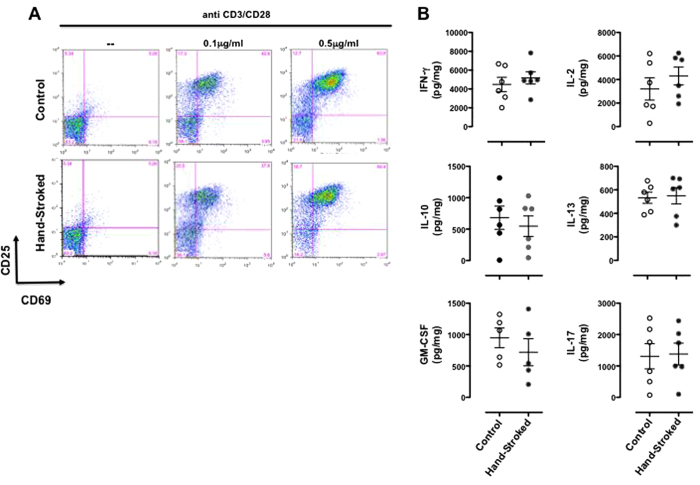
Massage-like stroking does not influence the activation profile of mature T cells. C57BL/6 male mice were subjected to massage-like therapy (hand-stroked) or left untouched (control) for 7 days and then analysed for their profile of T cell activation *in vitro*. (**A**) T cells were stimulated with the indicated concentrations of plate-bound anti-CD3 plus anti-CD28 for 16-18 hrs and then stained and analysed for their expression of CD25 and CD69 by FACS. (**B**) The plots show the levels of the indicated cytokines present in the supernatants of T cells stimulated with 0.5 μg/ml of plate bound anti-CD3 plus anti-CD28 for 22-24 hours. Values are expressed as mean ± S.E.M. and representative of 2 experiments, each involving 6 mice per group.

To assess if the boosting effects of massage-like therapy on T cell number were also accompanied by changes in their function, we performed some basic tests on T cell activation using plate-bound anti-CD3 plus anti-CD28 (anti-CD3/CD28) as stimulus. Stimulation of T cells from hand-stroked mice with suboptimal (0.1 μg/ml) or optimal (0.5 μg/ml) of anti-CD3/CD28 caused a concentration dependent increase in CD25 and CD69 (markers of T cell activation) expression that was comparable with that observed in control cells (Fig. 7A). In addition, analysis of cytokine levels in the supernatants of stimulated T cells showed no significant difference in the levels of classical Th1 (IFN-γ, IL-2 and GM-CSF), Th2 (IL-10 and IL-13), or Th17 (IL-17) cytokines between the two groups (Fig. 7B).

### Massage-like therapy reverses the immunosuppressive effects of hydrocortisone on thymocytes

**Figure 8:**
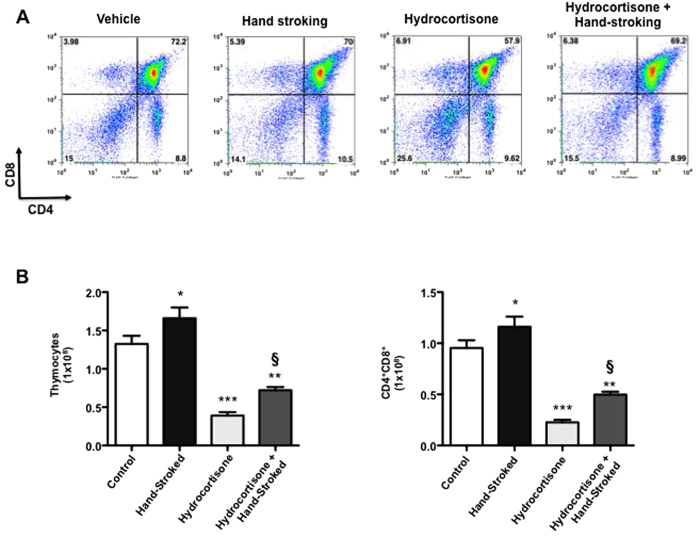
Massage-like stroking reverts the immunosuppressive effect of hydrocortisone on CD4+CD8+ double positive T cells. C57BL/6 male mice received an intraperitoneal injection of hydrocortisone (2.5 mg/mouse) or vehicle and then subjected to massage-like therapy (hand-stroked) or left untouched (control) for 7 days as indicated in the figure. At day 4 post hydrocortisone challenge T cell repertoire in the thymus was analysed. The panels in (**A**) show representative dot plots of the different subpopulations of thymocytes while (**B**) shows the total (left panel) or CD4^+^CD8^+^ double positive (right panel) thymocyte cell count. Values are expressed as mean ± S.E.M. and representative of 2 experiments, each involving 6 mice per group. * p < 0.05; ** p < 0.01; ***p < 0.001 *vs* vehicle control. § p < 0.05 *vs* hydrocortisone.

Clinical evidences suggest that massage is beneficial for the treatment of immunodepletion following chemotherapy^36^ or in patients suffering immunodeficiency disorders^3^. To recapitulate these findings we treated mice with hydrocortisone. This steroid is known to induce a transient immunosuppression through the induction of apoptosis of DP thymocytes (maximum at day 3/4 post injection) followed by recovery to its normal size and subset distribution a week following the treatment^37^,^38^. At day 4 post-treatment, about 30% of the DP thymocytes survived in the control group treated with hydrocortisone (Fig. 8). Conversely, mice that received hydrocortisone and massage-like therapy showed a reduction to only 60% (p < 0.05 *vs* control hydrocortisone treated mice). Similar differences, although not significant, were observed at day 8 when the thymus had fully recovered (data not shown).

## Discussion

The main aim of this study was to investigate the effect of massage-like stroking on the immune system in an experimental model. Both human (finger-driven) and non-human (brush stroke-driven) stroking showed a trend towards an increase in T cell number in lymphoid organs. However, only hand-delivered-stroking showed a statistical significant difference compared to control, reinforcing the hypothesis that the application of a controlled pressure might not be the sole parameter that contributes to the therapeutic effect of massage^17^. Other factors like the warmth or consistency of the contact area (the fingers in our case) might play a role.

Aiming to better understand the possible mechanism behind the observed differences in T cellularity, we first sought to investigate if this boosting action of massage on T cells was somehow linked to the hedonistic value of stroking and massaging on mood as it is often described for humans^17^. Our results suggested that this was not associated with significant difference in the levels of anxiety-like behaviour. There are many possible explanations for this discrepancy such as the length of the treatment itself not being enough to be “translated” into significant changes in anxiety-like behaviour (8 days as opposed to weeks) or, most likely, the absence of a modulatory effect of our massage-like paradigm on anxiety. In this regard it is interesting to note that studies addressing the handling of mice with tunnels have reported similar findings e.g. that handling does not always have an anxiolytic effect as this seems to be dependent on the strain of the mice, the experimental test used and, last but not least, the social interaction with other mice^39^,^40^.

These surprising results contradict our initial hypothesis that the positive emotional response elicited by the massage would be ultimately linked to its immunomodulatory effect and prompted us to explore other possible mechanisms. Previous studies performed in humans have suggested that massage could either enhance vagal activity *via* stimulation of pressure receptors that ultimately signal the limbic system^41^,^42^ or decrease the release of norepinephrine in the blood stream causing an overall down regulation of sympathetic activity^43^,^44^. Intriguingly, studies by our own lab and other research groups have shown sympathetic nerves that innervate lymphoid tissues as one of the major pathways by which the neuronal and immune system communicate to maintain body homeostasis^35^,^45^,^46^,^47^,^48^. Most importantly, evidence suggests that this homeostatic cross-talk is responsible for the “processing of information” such as behavioural conditioning or changes of external environmental factors and its translation into specific immune responses^32^,^49^.

Our analysis of the noradrenergic tone present in the thymus and spleen of stroked mice supported these findings and showed a drastic reduction in nerve density and noradrenaline content when compared to control mice. Thymic noradrenergic nerves originate primarily from the superior cervical and stellate ganglia and enter the thymus with large blood vessels, ending into the capsule and interlobular septa. From these vascular nerve plexuses, smaller vascular plexuses diverge into the cortex. The spleen instead receives a rich supply of sympathetic nerves primarily from the superior mesenteric and celiac ganglionic plexuses and its noradrenergic innervation is distributed extensively to various parts of the spleen including capsule, trabeculae, red pulp, and white pulp^47^,^50^,^51^. The functional significance of this intricate network is to provide both potentiation and inhibition of immune functions. In our studies, the decreased noradrenergic tone of both thymus and spleen caused a significant increase in T cell cellularity as it has been observed in other settings through pharmacological or surgical reduction of the noradrenergic tone^35^. This boost in T cell number was not accompanied by changes in TCR response *in vitro*, as shown by our tests on mature T cell activation, supporting the idea that the immunomodulatory effects we observed *in vivo* might be due to the lymphoid organ microenvironment, rather than a direct effect at the level of T lymphocyte gene expression e.g. to their capacity to expand and proliferate.

How does massage-like stroking decrease noradrenergic tone? Although we have not fully addressed this question, there is growing evidence in the literature supporting the concept of an interactive network between cutaneous nerves, the neuroendocrine axis and the immune system. Based on these theories, the skin can be genuinely considered a neuroimmunoendocrine organ that controls a wide variety of functions through the peripheral sensory nervous system, the autonomous nervous system, as well as the central nervous system (for an in depth review on the topic see^52^). This idea has been further substantiated by a recent novel study by Vrontou *et al.*^22^, that has identified unmyelinated C type sensory neurons that detect massage-like stroking on hairy skin in mice. These neurons, named MRGPRB4+, exclusively innervate hairy skin and have been shown to be closely related to the C-LTMRs found in humans^22^. Most strikingly, these fibres terminate in the substantia dorsal horn of the spinal cord with neurons^53^ that give projections to the insular cortex^54^, an area of the brain concerned with wellbeing and emotion^55^, but also shown to play a crucial role in the central autonomic network^90^. Sympathetic nerve responses to insular cortical stimulation are mediated by synapses within the lateral hypothalamic area and ventrolateral medulla^91^, the brain structures linked with immunomodulation^92^.

Notwithstanding the importance of fully investigating the cellular and molecular mechanisms behind the immunomodulatory function of massage, one of the major findings of this study is the confirmation in experimental animals of observations made in humans for the effectiveness of massage in the treatment of immunodeficiencies^3^,^56^,^57^,^58^. The concept that massage has ‘recovering’ properties is well known in the field of sport medicine where it has been shown that massage has an important immunoregulatory function after strenuous exercise^59^,^60^. More specifically, massage has been shown to favour recovery from the transient immunosuppressive state induced by exercise through release of more cells in the circulation and controlling the infiltration of inflammatory cells into the muscles^61^,^62^. Similarly, there are some evidences that massage may support the recovery of immune function during periods of immunosuppression, counteracting the loss of T cells in patients suffering from cancer and HIV infection^3^,^16^,^19^,^58^. Although peripherally comparable to these studies, our results support these findings and suggest that massage contribute to the maintenance of immunocompetence.

More investigations are required both at the experimental and clinical levels to fully appreciate the potential therapeutic application of massage therapy used as co-adjuvant to standard therapy. Nevertheless, we think our studies support innovative scientific views that stress the importance of considering the multiplicity of systems (system biology) that homeostatically regulate what is commonly known as “wellbeing”^63^. Indeed, our results support the concept that mechanisms regulating homeostasis^64^,^65^ are of fundamental importance for a better understanding of the causes of immune and inflammatory “dis-ease”- from the old-french “aise” and the latin “adjacens” (living close by).

## Additional Information

**How to cite this article**: Major, B. *et al.* Massage-like stroking boosts the immune system in mice. *Sci. Rep.* **5**, 10913; doi: 10.1038/srep10913 (2015).
